# A new approach for ratiometric *in vivo* calcium imaging of microglia

**DOI:** 10.1038/s41598-017-05952-3

**Published:** 2017-07-20

**Authors:** Bianca Brawek, Yajie Liang, Daria Savitska, Kaizhen Li, Natalie Fomin-Thunemann, Yury Kovalchuk, Elizabeta Zirdum, Johan Jakobsson, Olga Garaschuk

**Affiliations:** 1 Institute of Physiology II, University of Tübingen, 72074 Tübingen, Germany; 2 Laboratory of Molecular Neurogenetics, Department of Experimental Medical Science, Wallenberg Neuroscience Center and Lund Stem Cell Center, Lund University, Lund, 221 84 Sweden

## Abstract

Microglia, resident immune cells of the brain, react to the presence of pathogens/danger signals with a large repertoire of functional responses including morphological changes, proliferation, chemotaxis, production/release of cytokines, and phagocytosis. *In vitro* studies suggest that many of these effector functions are Ca^2+^-dependent, but our knowledge about *in vivo* Ca^2+^ signalling in microglia is rudimentary. This is mostly due to technical reasons, as microglia largely resisted all attempts of *in vivo* labelling with Ca^2+^ indicators. Here, we introduce a novel approach, utilizing a microglia-specific microRNA-9-regulated viral vector, enabling the expression of a genetically-encoded ratiometric Ca^2+^ sensor Twitch-2B in microglia. The Twitch-2B-assisted *in vivo* imaging enables recording of spontaneous and evoked microglial Ca^2+^ signals and allows for the first time to monitor the steady state intracellular Ca^2+^ levels in microglia. Intact *in vivo* microglia show very homogenous and low steady state intracellular Ca^2+^ levels. However, the levels increase significantly after acute slice preparation and cell culturing along with an increase in the expression of activation markers CD68 and IL-1β. These data identify the steady state intracellular Ca^2+^ level as a versatile microglial activation marker, which is highly sensitive to the cell’s environment.

## Introduction

Microglia are resident immune cells of the central nervous system (CNS) classically thought to mediate the innate defence responses against pathogens as well as brain injury^1, 2^. Recently, however, they were shown to contribute to many basic processes of brain development and homeostasis, such as neurogenesis and axonal growth, formation, remodelling and plasticity of synapses, modulation of neuronal activity via cytokine release and CNS vascularisation^2–4^. Furthermore, microglia likely play an important role in ageing. In the aged brain microglia are characterized by decreased process complexity and a reduced territory covered by the processes as well as increased expression levels of pro-inflammatory cytokines^5^. It has been suggested that ageing-induced microglial dysfunction might contribute to a reduced repair capacity in aged individuals thus promoting neurodegenerative diseases^6^. Finally, microglia is central to the development and progression of neurodegenerative diseases themselves and the microglial/immune response genes were recently discovered as potent risk modifiers in many neurodegenerative diseases^2, 7–11^. Under steady-state conditions microglia have highly ramified long motile processes, actively surveying the surrounding territory. The appearance of damage- (DAMPs) or pathogen-associated molecular pattern molecules (PAMPs) in the cell’s vicinity initiates activation of microglia, which is associated with a profound change in morphological appearance as well as functional properties of these cells. Depending on the strength of the DAMP/PAMP stimulus, microglial cells engage in different effector responses including cytoskeletal rearrangements, process extension, migration to the site of injury, enhanced phagocytosis as well as release of proinflammatory cytokines, nitric oxide (NO) and neurotrophic factors^2, 12^. Mounting *in vitro*/*in situ* data suggest that many of these effector responses are mediated by intracellular Ca^2+^ signals^13–18^, but our knowledge about *in vivo* Ca^2+^ signalling in microglia still remains rudimentary.

This is mainly due to the fact that microglia largely resisted all attempts to label them with small molecule as well as genetically-encoded Ca^2+^ indicators (GECI)^15, 19, 20^. Seifert *et al*. attempted to label microglia with a retrovirus encoding a GECI GCaMP2^15^. Because retroviruses only transduce dividing cells, the virus had to be injected 2 days after stab wound injury, triggering microglial proliferation. This approach enabled monitoring agonist-evoked microglial Ca^2+^ signals in brain slices and theoretically it can also be applied *in vivo*. However, it does not distinguish between different types of proliferating cells and stab wound-induced microglial activation might modify the functional properties of labelled cells^21^. At the same time we labelled *in vivo* microglia by mild electroporation, enabling high-resolution imaging of microglia in acute *in vivo* experiments^19^. The data obtained provided the first insight into *in vivo* Ca^2+^ signalling of microglia and have shown that in the healthy adult brain microglia is rather silent in terms of its somatic Ca^2+^ signalling. However, they promptly respond with large Ca^2+^ transients to damage of nearby cells^19^. Moreover, ageing and amyloid accumulation dramatically increased the incidence of somatic Ca^2+^ transients in cortical microglia^22^. Despite these encouraging results, electroporation technique cannot be widely used for analyses of microglial physiology because of several limitations: (i) it is very laborious since each cell has to be approached individually, (ii) it is not applicable in longitudinal experiments and (iii) it cannot be excluded that electroporation itself modifies the cell’s function. Very recently a GECI GCaMP5G was successfully expressed in the intact *in vivo* microglia^23, 24^. This allowed to record spontaneous and agonist-evoked Ca^2+^ transients in a group of neighbouring cells and to uncover synchronized Ca^2+^ transients in several lipopolysaccharide (LPS)-primed cortical microglia responding to focal laser injury as well as LPS-primed cortical microglia recorded during the bicuculline-induced epileptiform activity^24^. Conveniently, GCaMP5G-labelled microglia also expressed a red fluorescent protein tdTomato, allowing better visualization of cell’s morphology. The only drawback of this technique is a rapid bleaching of both dyes during the 20-min-long continuous imaging regime^24^.

So far, however, all techniques available for *in vivo* Ca^2+^ imaging of microglia were sensitive to transient changes in the intracellular Ca^2+^ concentration ([Ca^2+^]_i_) only and unable to detect prolonged sustained elevations of [Ca^2+^]_i_. On the other hand, *in vitro* data suggest that well known PAMPs, such as LPS, cause chronic elevations of [Ca^2+^]_i_ and that these chronic elevations are required for effector responses of microglia such as release of NO or certain cytokines and chemokines^13, 14^. To enable reliable *in vivo* measurements of steady state intracellular Ca^2+^ level as well as Ca^2+^ transients we chose to express in microglia our novel ratiometric GECI Twitch-2B^25^ by means of a microRNA-9-regulated lentiviral vector^26^.

## Results

### Design of the viral vector

**Figure 1:**
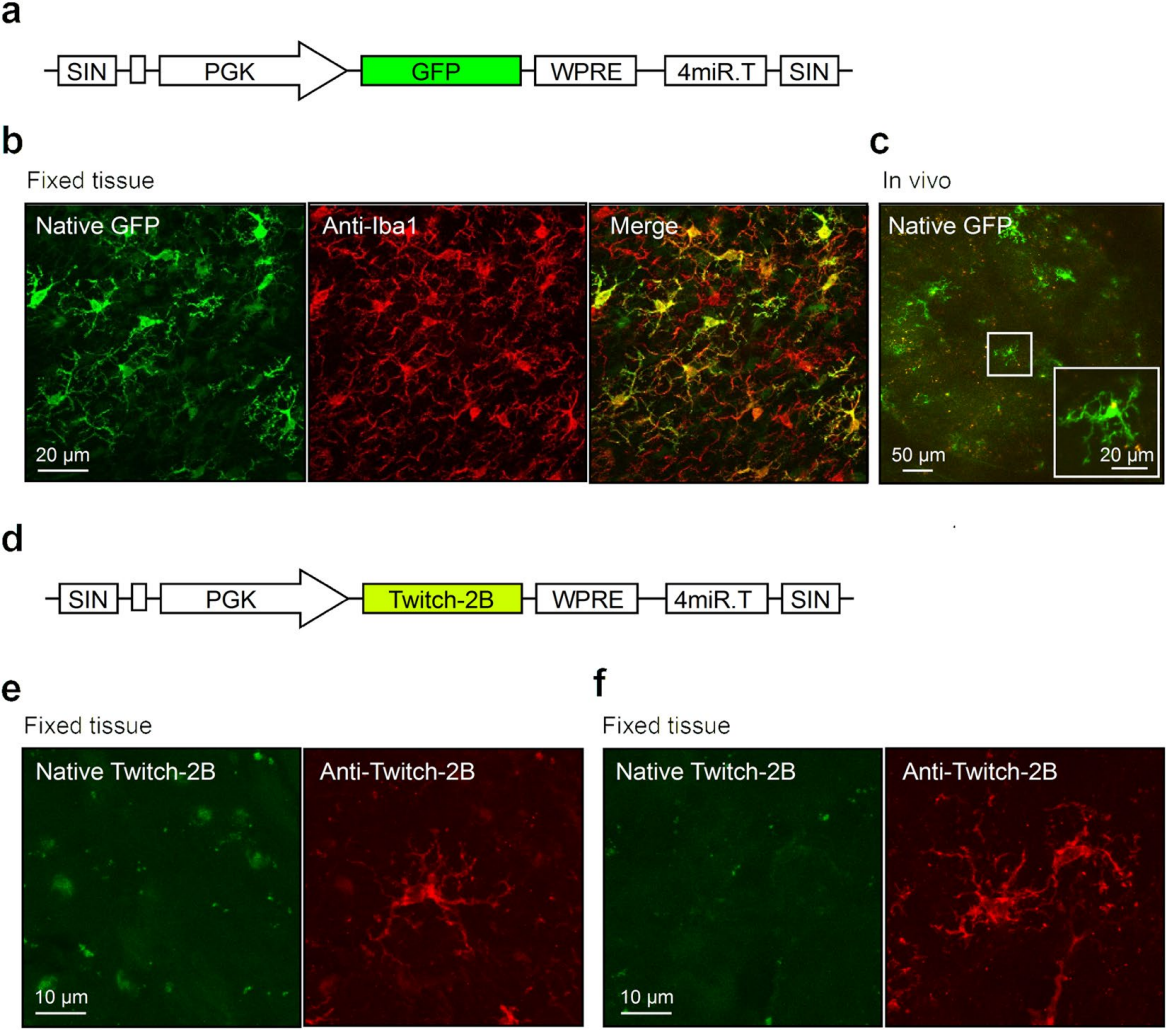
Expression of target genes (GFP or Twitch-2B) in LV.miR-9.T vector-transduced brain tissue. (**a**) Scheme of the LV.PGK.GFP.miR-9.T construct. (**b**) Maximum intensity projection (MIP) images (5–15 µm, step 1 µm) illustrating the identity of LV.PGK.GFP.miR-9.T-transduced cells (left) through immunofluorescent staining with an antibody against the microglial marker Iba1 (middle). The colour merged image is shown on the right. (**c**) Two-photon MIP *in vivo* image (18–27 µm, step 2 µm) of the transduced cells showing typical microglial morphology. Inset is a MIP image (3–21 µm, step 1 µm) showing a zoom-in of a rectangular square in the middle of the image. (**d**) Scheme of the LV.PGK.Twitch-2B.miR-9.T construct. (**e**,**f**) MIP images ((**e**) 0–19 µm, (**f**) 0–20 µm, step 1 µm) showing either native fluorescence of the cells transduced with LV.PGK.Twitch-2B.miR-9.T construct (left) or fluorescence of the same area labelled with an anti-GFP antibody recognizing Twitch-2B (right).

The use of microRNA-9-regulated vectors is based on the incorporation of complementary microRNA-9 target sites into the transgene cassette. This leads to degradation of the transgene messenger RNA specifically in cells expressing microRNA-9 (miR-9). As we have shown previously, rodent microglia lacks miR-9 expression, and therefore miR-9-regulated vectors can be used for selective labelling of microglia^26^. To begin with, we transduced cortical cells in adult mice with the original miR-9-regulated vector in which the expression of green fluorescent protein (GFP) was driven by the phosphoglycerate kinase (PGK) promoter (LV.miR-9.T construct^26^; Fig. 1a) as well as a similar sequence encoding Twitch-2B instead of GFP (Fig. 1d). As expected, GFP selectively and brightly labelled microglial cells (Fig. 1b,c), whereas Twitch-2B fluorescence was neither detected *in vivo* (not shown) nor in *ex vivo*, fixed tissue (Fig. 1e,f, left panels). Immunocytochemical labelling of this tissue with an antibody against Twitch-2B (anti-GFP; Fig. 1e,f, right panels) revealed that Twitch-2B expression was present albeit weak.

**Figure 2:**
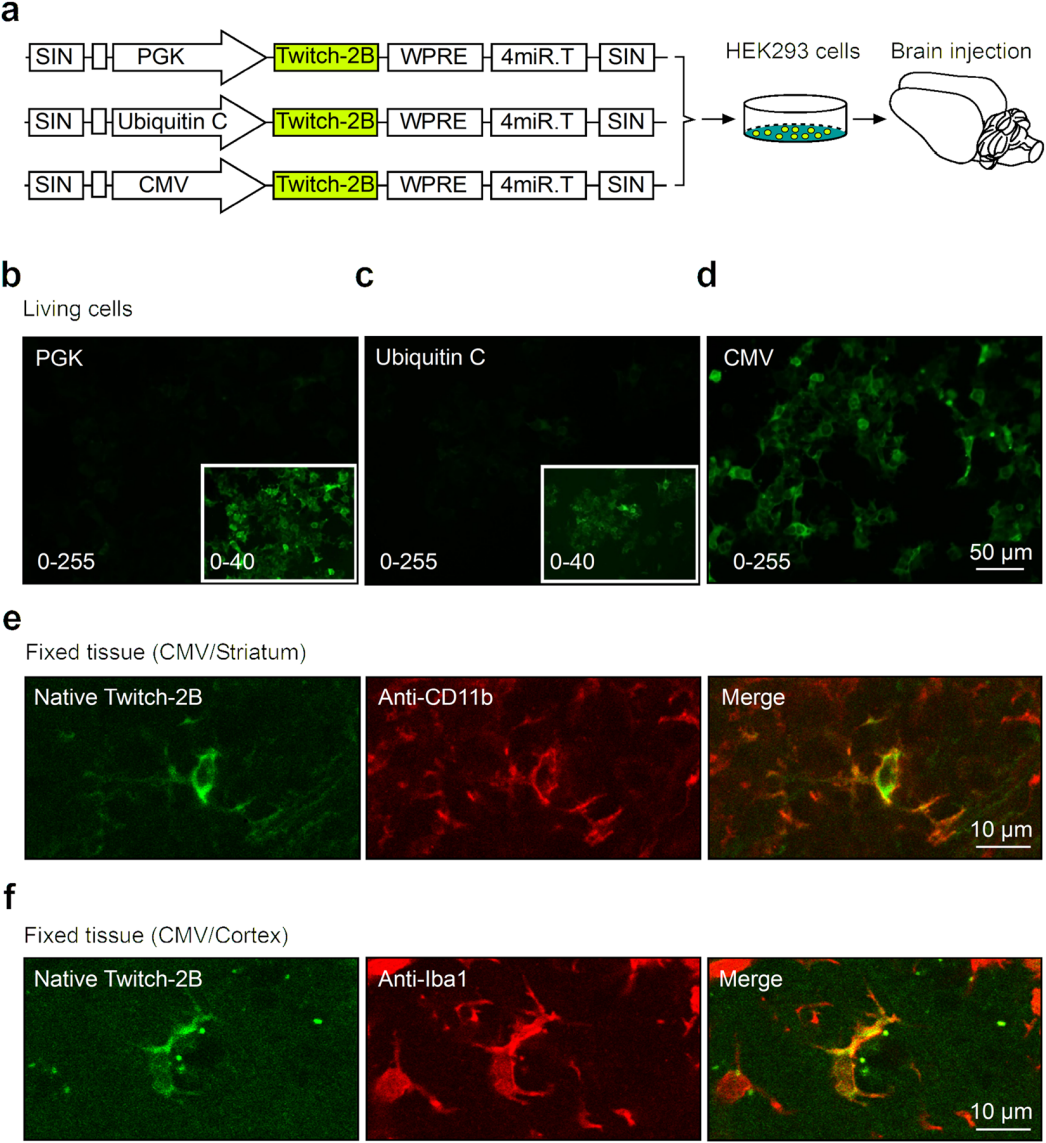
Optimization of the LV.Twitch-2B.miR-9.T vector. (**a**) Illustration of the experimental approach to test the efficacy of LV.Twitch-2B.miR-9.T vectors with different promoters. Lentiviral vectors were first compared in HEK293 cells *in vitro* and then tested in the brain *in vivo*. (**b**–**d**) Epifluorescence single-plane images of HEK293 cells transduced with different viral vectors shown in (**a**), taken with the same excitation light intensity and exposure time. Insets show the contrast-enhanced display of the same image. Numbers in the lower left corner show the range of display for each image. (**e**,**f**) Single-plane images of the fixed striatal (**e**) or cortical (**f**) tissue transduced *in vivo* with LV.CMV.Twitch-2B.miR-9.T viral vector. Note that Twitch-2B-expressing cells (left) are positive for CD11b (**e**, middle) and Iba1 (**f**, middle) and therefore are microglial cells. The colour merged images are shown on the right.

**Figure 3:**
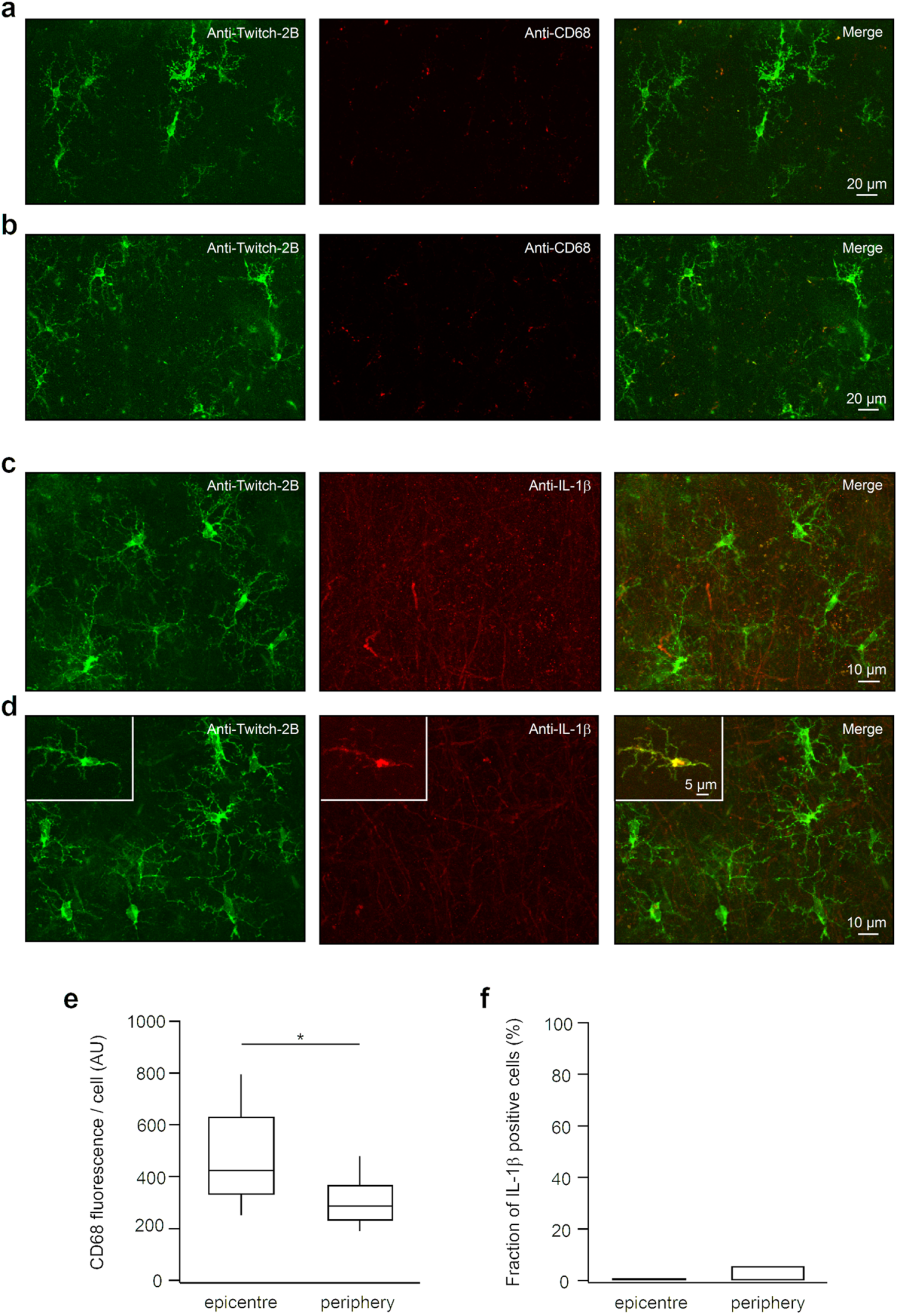
Post hoc analyses of activation markers expressed in Twitch-2B labelled microglia. (**a**,**b**) MIP images of fixed cortical tissue, transduced *in vivo* with LV.CMV.Twitch-2B.miR-9.T viral vector, taken in the epicentre (**a**; 7–59 µm, step 1 µm) and at the periphery (**b**; 4–44 µm, step 1 µm) of the injection site. Left: Twitch-2B positive microglial cells labelled with an anti-GFP antibody. Middle: the same field of view labelled with anti-CD68 antibody. Merged images are shown on the right. (**c**,**d**) MIP images of fixed cortical tissue (as above) taken in the epicentre (**c**; 2–28 µm, step 1 µm) and at the periphery (**d**; 1–33 µm, step 1 µm) of the injection site. Left: Twitch-2B positive microglial cells labelled with an anti-GFP antibody. Middle: the same field of view labelled with anti-IL-1β antibody. Merged images are shown on the right. Inset in (**d**) shows a rare Twitch-2B and IL-1β positive cell at the periphery of the injection site. (**e**) Summary box plot illustrating distributions of background-subtracted CD68 fluorescence per microglial cell in the epicentre (n = 100 cells in 3 mice) and at the periphery (n = 86 cells in 3 mice) of the injection site. Note that CD68 fluorescence is significantly higher in microglia in the epicentre compared to periphery (p < 0.001, Mann-Whitney test). (**f**) Summary bar graph showing the fraction of IL-1β positive cells in the population of Twitch-2B positive microglia in the epicentre (n = 31 cells in 4 mice) and at the periphery (n = 54 cells in 5 mice) of the injection site.

**Figure 4:**
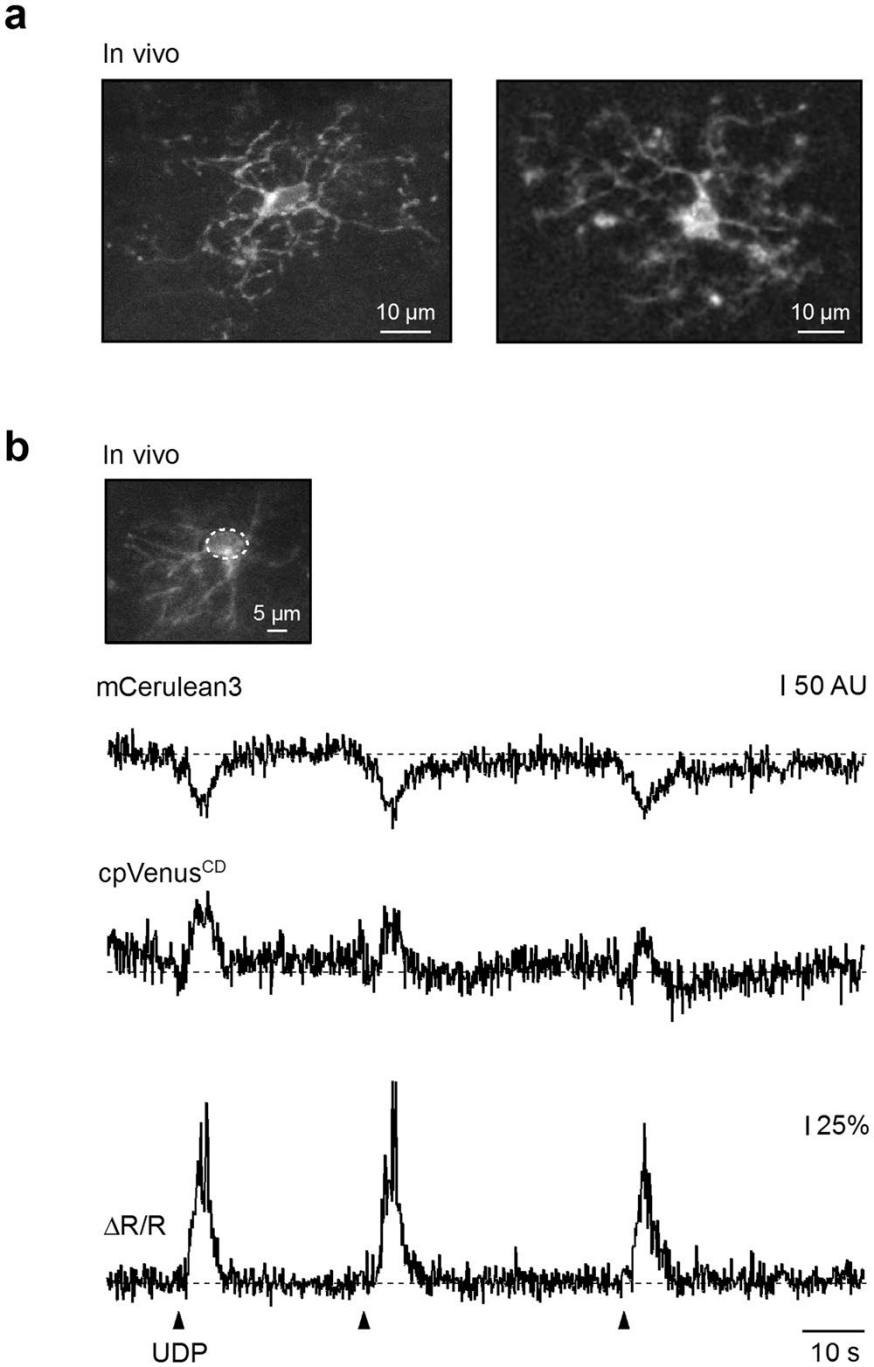
LV.CMV.Twitch-2B.miR-9.T vector enables ratiometric calcium imaging of microglia *in vivo*. (**a**) MIP images (left: 18–33 µm depth; right: 17–36 µm depth, step 1 µm) showing combined fluorescence of mCerulean3 and cpVenus^CD^ of cortical microglia *in vivo*. (**b**) Representative traces, recorded from the region of interest delineated in the inset (dashed line), showing mCerulean3 (top) and cpVenus^CD^ (middle) channels for three pressure applications of a P2Y receptor agonist UDP (1 mM in the application pipette, 200 ms). Inset: MIP image (117–153 µm depth, step 1 µm) of the recorded microglial cell. Bottom: trace showing the ΔR/R signal. Arrowheads indicate time points of UDP applications. Similar results were obtained in n = 5 cells (summarized in Fig. 5b).

**Figure 5:**
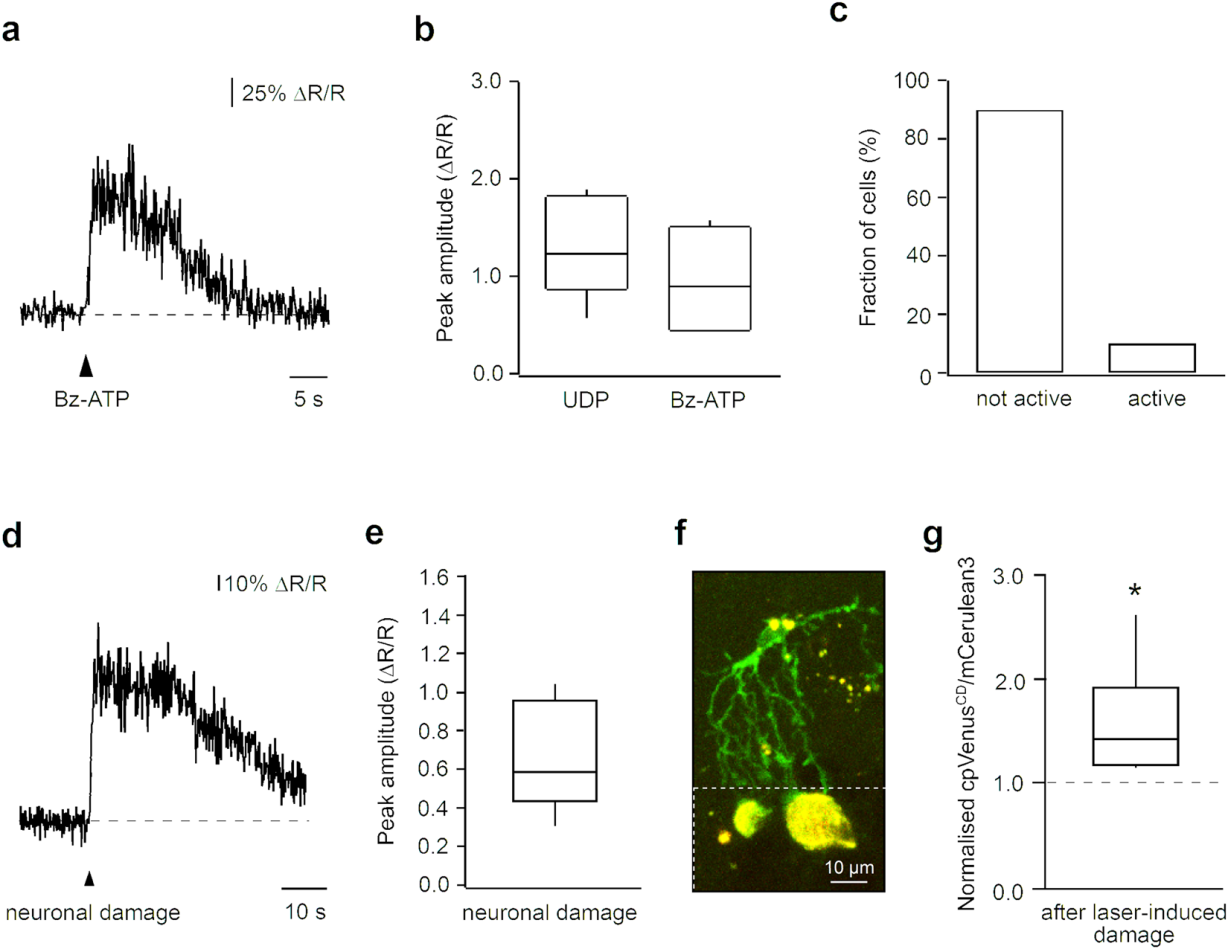
Versatility of the LV.CMV.Twitch-2B.miR-9.T vector for *in vivo* Ca^2+^ imaging of microglia. (**a**) Representative microglial Ca^2+^-transient evoked by pressure-application of a P2X receptor agonist Bz-ATP (1 mM in the application pipette, 100 ms). (**b**) Summary box plot showing peak amplitudes evoked by pressure-applications of UDP (1 mM, n = 5 cells) and Bz-ATP (1 mM, n = 5 cells) in microglial cells *in vivo*. (**c**) Bar graph showing the fraction of microglia with and without spontaneous Ca^2+^-activity over a 10-min-long imaging period (n = 10 cells). (**d**) Representative microglial Ca^2+^-transient evoked by damaging a neuron in cell’s vicinity (cell-to-cell distance 18 µm). (**e**) Box plot showing distribution of peak amplitudes of microglial Ca^2+^ transients evoked by damaging cells located 15–38 µm apart from the recorded microglia (n = 7 cell damage-induced transients, each caused by damaging a separate cell, recorded from 4 cells in 4 mice). The cell damage-induced Ca^2+^ transients were observed in 4 out of 5 microglial cells tested. (**f**) MIP image (56–84 µm depth, step 1 µm), taken 60 min after a laser induced injury of the cortical parenchyma, showing a Twitch-2B positive microglia (green, combined fluorescence of mCerulean3 and cpVenus^CD^) responding to tissue damage. Note that tissue autofluorescence (yellow) is increased in the laser-damaged area (indicated by a broken white line). (**g**) Box plot illustrating a significant increase in basal cpVenus^CD^/mCerulean3 fluorescence ratio in microglia after laser-induced damage compared to control conditions (median: 1.43, IQR: 1.19–1.93, p < 0.05; Wilcoxon Signed-rank test; n = 6 cells in 3 mice). The laser induced injury increased cpVenus^CD^/mCerulean3 fluorescence ratios in all microglial cells tested.

To improve the quality of *in vivo* labelling of microglia with Twitch-2B, we constructed two additional lentiviral vectors driving Twitch-2B expression by different internal promoter sequences: human ubiquitin C promoter or cytomegalovirus (CMV) promoter (Fig. 2a) and tested them in Human Embryonic Kidney (HEK) 293 cells. Compared to expression from the PGK promoter (Fig. 2b), the ubiquitin C promoter showed a similar performance whereas the CMV promoter was able to drive much stronger expression of Twitch-2B (Fig. 2c,d). The latter construct proved to be efficient also *in vivo*, labelling CD11b- and Iba1- (ionized calcium-binding adapter molecule 1) positive microglial cells in the striatum and in the cortex (Figs 2e,f and 3–5). Based on these data we chose the miR-9-regulated vector with CMV promoter for *in vivo* labelling of microglia with Twitch-2B.

### *In vivo* functional properties of Twitch-2B-expressing microglia

Injection of the Twitch-2B-encoding viral particles into the mouse cortex successfully labelled microglia in the surrounding tissue (Fig. 3). The labelled cells could be visualized up to the depth of 330 µm below the pial surface (Supplementary Fig. S1), but alike the *in vivo* labelling with GFP (Fig. 1c), the Twitch-2B-labelled tissue had a patchy appearance providing rather a collection of individual labelled cells than a labelled microglial network. Post-hoc immunohistochemical quantification of the transduced microglial population revealed that in the epicentre of the injection area 4.42–21.84% of the Iba1-positive microglia were also positive for Twitch-2B. At the periphery of the injection area (>150 µm distance from epicentre) this value amounted to 3.65–19.26%. In many cases we also observed Twitch-2B-labelled neurons and astrocytes at the epicentre of an injected area, which however could be clearly distinguished from microglia based on their morphology (not shown). On average, in the epicentre of the injection area only 36.58% (interquartile range (IQR): 30.6–42.15%; n = 37 fields of view in 8 mice) of Twitch-2B-positive cells were also positive for Iba1, and therefore microglia. This nonspecific labelling was most probably due to the strong expression of our construct in these cells, so that endogenous miR-9 was insufficient for degradation of the entire transgene messenger RNA. This finding precluded further attempts to increase the expression rate of the transgene and prompted us to work slightly off the centre of the injected area (>150 µm from the injection site), where microglia represented the majority of labelled cells (62.45%, IQR: 47.39–68.39%; n = 55 fields of view in 8 mice).

Further, we examined whether the labelling procedure per se produced an activation of microglial cells. To this end, during post-hoc analyses Twitch-2B labelled cells in the epicentre and at the periphery of the injection area were stained with antibodies against the known markers for activated microglia CD68 and IL-1β (Fig. 3a–d). Microglia in the centre of the injection area showed slightly but significantly higher expression levels of CD68 compared to microglia at the periphery (Fig. 3e), whereas no IL-1β-positive cells were found in the centre of the injection area (Fig. 3c,f). At the periphery, 3 out of 54 cells (n = 5 mice; Fig. 3f) were positive for IL-1β (see inset in Fig. 3d). Together, these data point towards an absence of an overt labelling-induced inflammation both in the epicentre and at the periphery of the injection area. Note also the similarity of the CD68 expression levels in the peripheral Twitch-2B labelled cells, the only cells used for functional measurements in this study, and intact microglia (Supplementary Fig. S3e), imaged under similar experimental conditions.

When imaged *in vivo* in anaesthetized mice, the Twitch-2B-labelled microglial cells had a ramified morphology (Fig. 4a) and, as expected for a FRET (Förster resonance energy transfer)-based ratiometric indicator, responded to brief pressure applications of P2Y receptor agonist UDP^19^ with a transient decrease in the donor and a transient increase in the acceptor fluorescence (Fig. 4b, upper and middle traces), indicating a Ca^2+^-mediated increase in FRET between the two fluorophores. This resulted in large reproducible changes in the cpVenus^CD^/mCerulean3 ratio, expressed as ΔR/R (Fig. 4b, lower trace). Conveniently, the obtained ratio traces were insensitive to pressure application-induced small tissue movements, focus drifts or slight bleaching of the dye (Fig. 4b), all of these being the known advantages of ratiometric indicators.

To test whether the wavelength-dependent light scattering might impact the cpVenus^CD^/mCerulean3 ratios measured *in vivo* under our experimental conditions (0–330 µm depth), we plotted the resting ratios measured in different cells vs. the depth of the cell below the dura surface. We have not observed any correlation between the cell’s depth and the cpVenus^CD^/mCerulean3 ratio both for chronic and acute preparations (Supplementary Fig. S1), and therefore concluded that differential light scattering had little impact on the ratios measured in this study.

Twitch-2B-labelled microglia also readily responded to an agonist of ionotropic P2X receptors 3′-O-(4-benzoyl)benzoyl ATP (Bz-ATP; Fig. 5a). On average, the amplitudes of UDP- and Bz-ATP-evoked responses were large (Fig. 5b), comparable to (or even larger than) the ones recorded with a “golden standard” small molecule Ca^2+^ indicator Oregon Green 488 BAPTA-1 (OGB-1)^19^. Next, we revisited other physiological properties of *in vivo* microglia, originally recorded in electroporated (i.e. potentially damaged^19^) cells. Consistent with the data obtained previously, intact Twitch-2B-expressing microglia showed very little spontaneous activity under steady-state conditions (Fig. 5c), but vividly responded with large Ca^2+^ transients to the damage of individual cells (performed as described in ref. 19, see Methods) in its vicinity (Fig. 5d,e). Similarly, steady-state cpVenus^CD^/mCerulean3 ratios of microglia increased significantly 1–2 min after laser-induced injury of the brain parenchyma in their vicinity (Fig. 5f,g).

Thus, miR-9-regulated expression of the FRET-based GECI Twitch-2B enables labelling of microglial cells as well as monitoring their spontaneous, cell/tissue damage- and agonist-induced Ca^2+^ signals *in vivo*.

### Monitoring steady-state calcium levels in microglia

**Figure 6:**
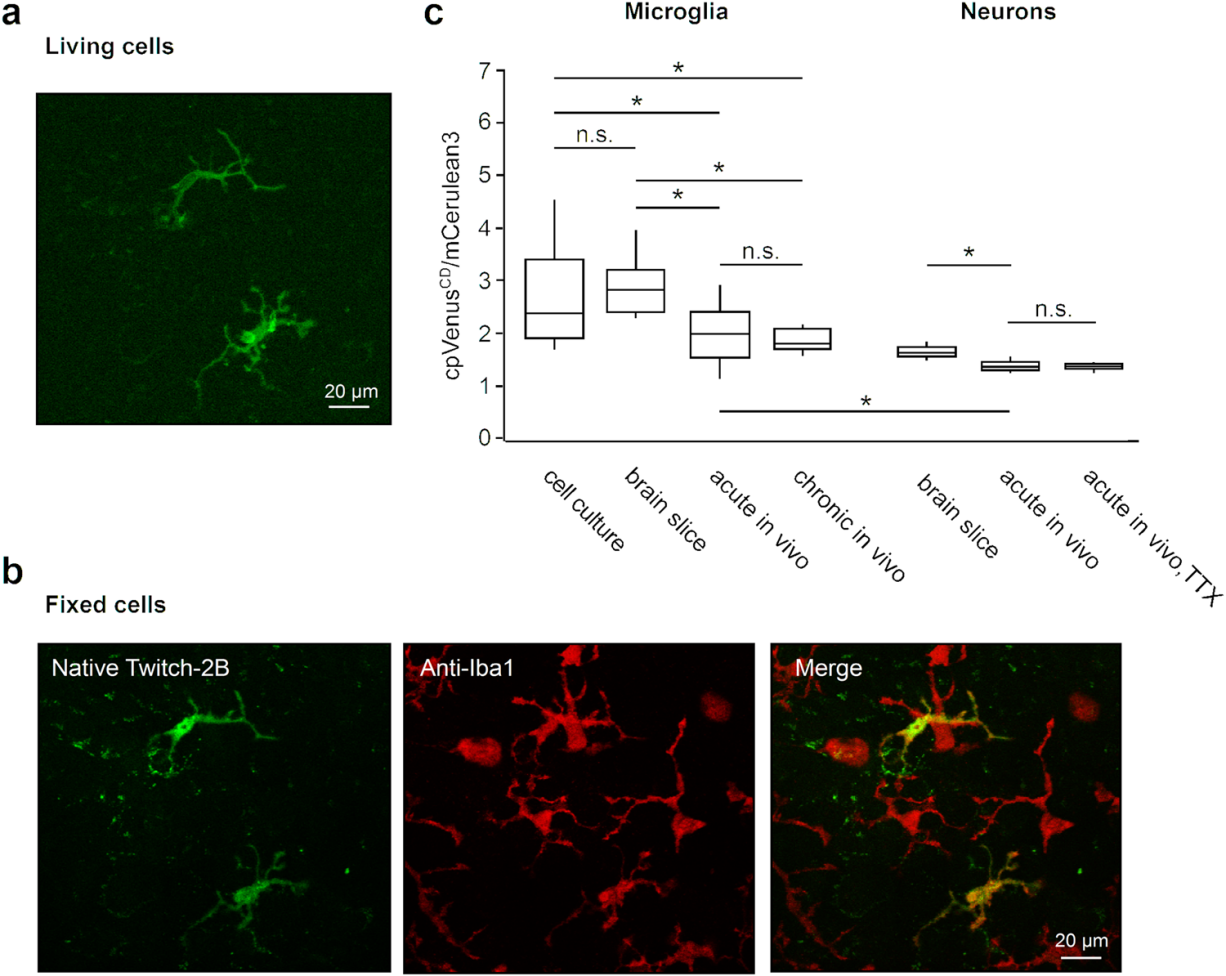
Use of the LV.Twitch-2B.miR-9.T vector enables estimation of basal Ca^2+^ levels in microglia. (**a**) MIP image (6–15 µm depth, step 1 µm; mCerulean3 channel) showing Twitch-2B-positive ramified microglial cells in culture. (**b**) MIP images of the same preparation after fixation and labelling with an anti-Iba1 antibody. Left: native fluorescence of Twitch-2B (combined signal from mCerulean3 and cpVenus^CD^); middle: fluorescence of Alexa Fluor 594 bound to anti-Iba1 antibody; right: a colour merged image of the two channels. (**c**) Box plot showing cpVenus^CD^/mCerulean3 fluorescence ratios of cortical microglia and neurons under different experimental conditions. When compared to ramified microglia in acute *in vivo* preparations (n = 32 cells), microglia in primary cell culture (n = 50 cells) and acute brain slices (n = 25 cells) show elevated cpVenus^CD^/mCerulean3 ratios (p < 0.0001 for acute *in vivo* vs. brain slice and 0.007 for acute *in vivo* vs. cell culture, Kruskal-Wallis test followed by post hoc Dunn-Sidak test). The same is true for comparison with microglia in chronic *in vivo* preparations (n = 15 cells; p < 0.0001 for chronic *in vivo* vs. brain slice and 0.006 for chronic *in vivo* vs. cell culture, Kruskal-Wallis test with post hoc Dunn-Sidak correction). In acute preparations, microglia exhibit significantly higher Ca^2+^-levels than cortical layer 2/3 neurons labelled with a AAV.synapsin1.Twitch-2B vector (n = 147 cells; p < 0.0001, Mann-Whitney test). TTX (2 µM) has no effect on basal cpVenus^CD^/mCerulean3 ratios of cortical neurons (n = 202 cells; p = 0.343, Kruskal-Wallis test with post hoc Dunn-Sidak correction). Note that neurons in acute brain slices (n = 601 cells) have slightly but significantly higher Ca^2+^-levels compared to those in acute *in vivo* preparations (p < 0.0001, Kruskal-Wallis test with post hoc Dunn-Sidak correction).

Next, we determined basal levels of cpVenus^CD^/mCerulean3 ratio at different experimental conditions both *in vitro* and *in vivo* (Fig. 6) using the LV.Twitch-2B.miR-9.T vector under control of the CMV promoter. The majority of microglial cells imaged in cell culture had a ramified morphology (Fig. 6a), and all of them were identified as Iba1-positive by the post-hoc immunocytochemistry (Fig. 6b). Cultured microglial cells had heterogeneous, but generally high steady-state Ca^2+^ levels with median ratio of 2.38 (IQR: 1.92–3.40; Fig. 6c). Similar basal ratios were measured using the original PGK promoter instead of the CMV promoter (Supplementary Fig. S2).

Subsequently, we measured basal levels of cpVenus^CD^/mCerulean3 ratio in microglia from acute cortical slices (50–100 µm under the slice surface), prepared from brains injected *in vivo* with LV.Twitch-2B.miR-9.T vector driven by a CMV promoter (Fig. 6c). The basal levels of cpVenus^CD^/mCerulean3 ratio in microglia from acute brain slices (2.82; IQR 2.42–3.20) were similar to those of primary microglia, but significantly higher than ratios measured both in acute (2.01; IQR 1.57–2.44) and chronic (after implantation of a chronic cranial window; 1.80; IQR 1.72–2.10) *in vivo* experiments (Fig. 6c). Moreover, basal levels of cpVenus^CD^/mCerulean3 ratio in *in vivo* microglia were slightly, but significantly higher than the ones recorded in cortical neurons (1.36; IQR 1.30–1.45). The neurons were labelled with an adeno-associated virus encoding the expression of Twitch-2B under control of the synapsin promoter and recorded in a separate series of experiments under similar experimental conditions. The ratio values were recorded in the absence and in the presence of the blocker of voltage-gated sodium channels tetrodotoxine (TTX; topical application^27^) to compare the basal and the resting (in the absence of action potential firing) neuronal cpVenus^CD^/mCerulean3 ratios (Fig. 6c). Preparation of acute brain slices clearly damaged cortical neurons on the slice surface (ratios up 7.67, not shown), but caused only a slight increase in basal ratios of neurons located ≥50 µm below the slice surface (Fig. 6c). Thus, compared to acute *in vivo* conditions, preparation of brain slices causes on average a 19.8% increase in neuronal basal cpVenus^CD^/mCerulean3 ratio (median ratio 1.63; IQR 1.55–1.73), suggesting that at the age tested (4-month-old) the neurons in brain slices show a slight but persistent impairment of their intracellular Ca^2+^ homeostasis compared to *in vivo* conditions. For microglia the difference in the basal cpVenus^CD^/mCerulean3 ratio between acute *in vivo* recordings and brain slices was 41.0% and thus twice as high as for neurons.

To test whether increased microglial cpVenus^CD^/mCerulean3 ratios measured in reduced preparations reflect activation of these cells, we compared the expression of microglial activation markers (CD68 and IL-1β) between cultured microglia and *in vivo* perfused and fixed brain tissue. Our results show that cultured microglia express significantly higher levels of the phagocytosis marker CD68 compared to *ex vivo* microglia (Supplementary Fig. S3a,b,e). Furthermore, 47.80% (IQR 39.29–54.93%) of cultured microglia were positive for IL-1β, a proinflammatory cytokine known to be released by activated microglia^14^, whereas no IL-1β-positive cells could be detected in the *ex vivo* fixed brain tissue (Supplementary Fig. 3c,d,f). These results show that microglia in cell culture are characterized by a more activated phenotype compared to their *in vivo* counterparts.

Taken together, our data show that *in vivo* the steady-state Ca^2+^ levels in microglia are low, but higher than those in neurons. Moreover, microglial cells react to changes in their environment caused by isolation/culturing or slice preparation with a further long-lasting increase in [Ca^2+^]_i_ and a significant upregulation of activation markers.

## Discussion

This study describes a new tool for assessing functional properties of microglia in the intact *in vivo* brain. It utilizes a FRET-based ratiometric genetically-encoded Ca^2+^ indicator with improved brightness and sensitivity and allows for the first time to measure both transient and sustained elevations of [Ca^2+^]_i_ in microglia. It has to be noted, however, that the approach used is not genuinely cell-type specific. Its cell-type specificity depends on the amount of endogenous miR-9 in relation to the amount of mRNA encoded by the viral construct. According to our data, the need of strong transgene expression for FRET-based *in vivo* imaging, where some photons are inevitably lost due to light splitting into two channels and light scattering, likely overwhelms the degradation capacity of miR-9 in some neurons and astrocytes resulting in a decrease of the cell-type specificity of the method. Future efforts in either providing more light-sensitive imaging techniques or brighter ratiometric Ca^2+^ indicators, enabling the *in vivo* use of the weaker (e.g. PGK) promoter, might help to circumvent this problem. At present, we chose to analyse the cells at the periphery of the injected area, where the majority of Twitch-2B-expressing cells are Iba1 positive and therefore microglia, and to use prominent morphological differences for further differentiation between the different cell types. Furthermore, because our technique does not provide the labelling of the entire microglial network, it is best suited for analysing (patho)physiological properties of many individual cells. The similarity of *in vivo* functional properties of the cells analysed in this study and those deliberately chosen previously for single-cell electroporation analyses^19^ suggests that cells studied are representative for the entire microglial population and argues against the assumption that the miR-9-regulated vectors target a functionally distinct subpopulation of microglial cells. In the future, current limitations might be surmounted by crossing transgenic animals expressing the floxed variant of Twitch-2B or another type of FRET-based Ca^2+^ indicator with microglia-specific Cre-driver mice (e.g. CX3CR1^CreER^ mice^28^), given that the resulting amount of Ca^2+^ indicator in microglia will be sufficient for *in vivo* imaging. However, the latter technique requires lengthy and laborious animal breeding and becomes even more tedious if consequences of genetic modifications have to be studied in triple or quadruple transgenic mice. In contrast, with the help of viral vectors, the indicator can be easily expressed in any area of the brain, at any developmental stage and in any mouse strain. Although currently used in mice, we anticipate that the same or similar vectors will also work in other rodent species (see ref. 26).

Our *in vivo* data obtained in intact Twitch-2B-expressing microglia confirmed our earlier findings in cells loaded with a small molecule Ca^2+^ indicator OGB-1 by means of single-cell electroporation^19^. Thus, intact microglial cells rarely showed spontaneous Ca^2+^ transients, but vividly responded with large Ca^2+^ transients both to local applications of agonists of either ionotropic or metabotropic P2 receptors and to a local cell damage in the microglia’s vicinity. Similar data were recently obtained in a newly generated mouse line selectively expressing a genetically-encoded Ca^2+^ indicator GCaMP5G in microglia^24^.

At the same time, the use of a ratiometric Ca^2+^ indicator revealed a significant difference in steady-state Ca^2+^ levels of microglia in different preparations. In mice implanted with a chronic cranial window and imaged more than three weeks after window implantation microglial steady-state Ca^2+^ levels were low and extremely homogeneous. Still, they were significantly higher than steady-state Ca^2+^ levels in resting neurons, showing that microglia have a different set point of cytosolic [Ca^2+^]_i_. Interestingly, similar trend was recently observed for cortical astrocytes^29^, suggesting that this property might be common for neuroglia. Microglial steady-state Ca^2+^ levels were still low in acute *in vivo* preparations (similar to the ones used for imaging of OGB-1-labelled microglia^19^), but the values obtained were more heterogeneous raising a suspicion that some microglial cells might react to a minor damage in their vicinity with a sustained increase in the steady-state Ca^2+^ level. This suspicion was further substantiated by imaging microglial cells in acute brain slices and cell cultures. In these preparations ramified microglial cells consistently showed significantly higher and extremely heterogeneous steady-state Ca^2+^ levels. This finding adds a new facet to our knowledge about microglial (patho)physiology by showing that changes in the environment of ramified microglia translate into sustained changes in microglial [Ca^2+^]_i_. Based on the data obtained, we hypothesize that this response is graded with more damage to the environment or more microglial activation causing higher increases in basal microglial [Ca^2+^]_i_. In fact, this concept is known for a long time for the strong, lipopolysaccharide-induced activation of cultured microglia^13, 14^, and is now extended by our data to ramified cells.

Different steady-state Ca^2+^ levels observed in this study may cause a differential activation of transcription factors^30^ and, as a consequence, a differential expression of the downstream effector genes. Indeed, the Ca^2+^/calcineurin-activated isoforms of the nuclear factor of activated T cells (NFAT) were recently shown to regulate the phenotype of cultured microglia^31, 32^, and many microglial genes exhibiting low levels of expression *in vivo* were found to be markedly upregulated after 7 days in culture^33^. Moreover, strong upregulation of IL-1β expression in cultured microglia suggests that besides regulation of gene expression via NFAT- and possibly also CREB-regulated pathways^34, 35^, the sustained increase in [Ca^2+^]_i_ is very likely to cause the activation of the NLRP3 inflammasome and thereby an increased production of pro-inflammatory cytokines IL-1β and IL-18^17, 36^ in parallel to NFAT-mediated upregulation in the production of IL-6 and TNF-α^32^. Finally, the sustained increase in [Ca^2+^]_i_ probably impacts other Ca^2+^-sensitive signalling pathways in microglia such as purinergic signalling, pathogen-directed migration and phagocytosis^14, 32, 37^.

## Methods

### Viral vectors and transduction of cells in culture

The structure of the starting lentiviral vector (LV.GFP.miR-9.T) has been described elsewhere^26^. Briefly, the four target sites of miR-9 5′-TCATACAGCTAGATAACCAAAG-3′ were cloned immediately downstream the woodchuck hepatitis virus post-transcriptional response element (WPRE) of a third-generation lentiviral vector. In the starting lentiviral vector, GFP was driven by the PGK promoter derived from the phosphoglycerate kinase housekeeping gene. We replaced GFP by Twitch-2B between the restriction sites BamHI (upstream) and SalI (downstream), generating LV.Twitch-2B.miR-9.T. For comparison of lentiviral vectors with different promoters, PGK promoter was replaced by either cytomegalovirus (CMV) promoter or human ubiquitin C promoter between EcoRI (upstream) and BamHI (downstream) restriction sites. Cell-free supernatants containing viral particles were produced by transient transfection of HEK293T packaging cells with the lentiviral construct and packaging plasmids (psPAX2 and pMD2G) as described previously^38^. After 48 h the virus-containing culture supernatant was collected, filtered through a 0.45 µm pore-sized filter and concentrated by centrifugation at 27,000 rpm for 2 h at 4 °C by using Thermofisher WX Ultra80 centrifuge (Waltham, MA, USA). Pellets were re-suspended in phosphate buffered saline (PBS) and stored at −80 °C. Viral solutions with a titer higher than 10^8^ colony forming units/ml were used in this study. HEK293 cells were transduced with viral solutions of the same titer diluted 1:100. 48 h later, cells were fixed with 4% PFA for 10 minutes, and rinsed with PBS for observation under an epifluorescence microscope. All cells were imaged with the same fluorescence intensity and exposure time.

### Mice

Two to six months old C57BL/6 mice of either sex were used in this study. All experimental procedures involving the handling and use of mice were performed in accordance with institutional animal welfare guidelines and were approved by the state government of Baden-Württemberg, Germany. Mice were housed under a 12 h light/dark cycle and had free access to food and water.

### *In vivo* application of viral vectors

For *in vivo* labelling of microglia, we used the above mentioned lentiviral vector inducing Twitch-2B expression under the control of the CMV promoter in a miR-9 dependent manner. For labelling of layer 2/3 neurons, we used an adeno-associated virus driving Twitch-2B expression under the control of the synapsin promoter (Penn Vector Core, Philadelphia, PA, USA).

Mice were anaesthetized by intraperitoneal (i.p.) injections of ketamine (80 µg/g body weight (BW); Fagron, Rotterdam, The Netherlands) and xylazine (4 µg/g BW; Sigma-Aldrich, St Louis, USA). Anaesthetic depth was monitored by the toe pinch reflex throughout the surgery and additional ketamine (40 µg/g BW) and xylazine (2 µg/g BW) was injected when necessary. Alternatively a combination of Fentanyl (0.05 mg/kg BW; Albrecht GmbH, Aulendorf, Germany), Midazolam (5.0 mg/kg BW, Hameln pharma plus GmbH; Hameln, Germany), and Medetomidin (0.5 mg/kg BW; alfavet Tierarzneimittel GmbH, Neumünster, Germany) was used. A small drill hole was made into the skull over the injection site, exposing the brain. A pulled glass pipette (tip diameter ~30 µm) was loaded with viral solution and then lowered into the brain to the appropriate coordinates (striatum: anteroposterior: 0.86 mm; lateromedial: 2 mm; dorsoventral: −2.8 mm; somatosensory cortex: anteroposterior: −2 mm; lateromedial: 2.0 mm; dorsoventral: −0.8 and −0.4 mm, with a glass pipette angled at 40 degree; all values are given relative to the bregma). The viral construct was injected into the somatosensory cortex tissue using a two-step procedure, in which 0.5 µl of the viral solution was injected at a depth of 0.51 mm and another 0.5 µl at 0.26 mm with an application speed of 200 nl per min.

### Immunohistochemistry

Two-three weeks after viral injection, mice were processed for histological assessment. Mice were transcardially perfused with PBS followed by 4% formaldehyde in PBS. Brains were dissected, post-fixed in 4% formaldehyde in PBS overnight, and cryoprotected in PBS containing 25% sucrose (overnight, 4 °C). Brain tissue was embedded in Tissue Tek (Sakura; Staufen, Germany) and stored at −80 °C until further use. Brains were cut into 40 or 50 μm thick sagittal slices with a cryostat (CM1950; Leica, Wetzlar, Germany). Slices were first screened for Twitch-2B-labelled cells with an epifluorescence microscope and were then treated with a blocking solution (5% goat or donkey serum and 1% Triton X-100 in PBS) for 1 h. After blocking, slices were incubated overnight with primary antibodies. Next, the sections were rinsed in PBS three times for 10 min and incubated with Alexa Fluor 594- or Alexa Fluor 488-conjugated secondary antibodies (1:1,000 in PBS + 2% bovine serum albumin; Invitrogen, Grand Island, NY, USA) for 2 h in the dark. Finally, the sections were washed three times in PBS, transferred to Superfrost Plus charged glass slides (Langenbrink, Emmendingen, Germany) and mounted in Vectashield Mounting Medium (Vector Laboratories; Burlingame, CA, USA). The following primary antibodies were used: rabbit or goat polyclonal anti-GFP for Twitch-2B staining (1:500 or 1:2500; Rockland, Limerick, PA, USA), anti-Iba1 (1:500; Wako, Neuss, Germany), anti-CD11b (1:500; AbD SeroTec, Oxford, UK), anti-IL1β (1:200; R&D Systems, Minneapolis, MN, USA), and anti-CD68 (1:1000; AbD SeroTec, Oxford, UK).

Immunostained brain slices were imaged using an Olympus Fluoview 300 laser scanning microscope (Olympus, Tokio, Japan) coupled to a MaiTai mode-locked laser operating at 690–1040 nm wavelength (Spectra Physics, Santa Clara, CA, USA). Alexa Fluor 594 and Alexa 488 were excited at 800 nm, and the fluorescence signals were separated using a 570 nm dichroic mirror and 536/40 nm bandpass as well as 568 nm longpass filters. GFP and Twitch-2B were excited at 890 nm and the fluorescence signals were collected from the short-pass channel of the 570 nm dichroic mirror.

### Preparation of acute cranial window

Three to four weeks after the viral injection into the somatosensory cortex (coordinates see above), mice were anaesthetized using isoflurane (2–2.5% in O_2_ for induction, 0.8–1.50% in O_2_ for surgery and imaging; CP-Pharma, Burgdorf, Germany) and placed on a heating pad. During the experiment, body temperature was monitored and maintained between 36 and 37 °C. After subcutaneous injection of lidocaine (2%, AstraZeneca, Cambridge, UK), the skin above the skull was removed. A recording chamber with a central hole was glued to the exposed skull using cyanoacrylate glue (UHU, Bühl, Germany). The skull was gently thinned using a dental drill. Thereafter, the mouse was transferred to the imaging setup. The recording chamber was continuously perfused with pre-warmed (36 °C) extracellular solution containing 125 mM NaCl, 4.5 mM KCl, 26 mM NaHCO_3_, 1.25 mM NaH_2_PO_4_, 2 mM CaCl_2_, 1 mM MgCl_2_ and 20 mM glucose, pH 7.4 when bubbled with 95% O_2_ and 5% CO_2_. A small craniotomy with a size of 0.5 × 1 mm was performed above the injection site using a thin 30 G syringe needle. Care was taken not to damage the dura.

### Preparation of acute brain slices

Animals were decapitated under deep CO_2_ anaesthesia. The brain was quickly dissected and transferred into ice-cold extracellular solution (composition as above but with 0.5 mM CaCl_2_ added). Coronal brain slices (300 μm) were cut on a vibratome (VT1200 S; Leica, Wetzlar, Germany). For recordings, slices were placed into a recording chamber on the stage of the microscope and continuously perfused with pre-warmed extracellular solution (composition see above).

### Implantation of chronic cranial window

A chronic cranial window was installed over the injection site as described previously^38^. Briefly, mice were anaesthetized with ketamine/xylazine (80/4 µg/g BW) or a combination of Fentanyl (0.05 mg/kg BW; Albrecht GmbH, Aulendorf, Germany), Midazolam (5.0 mg/kg BW, Hameln pharma plus GmbH; Hameln, Germany), and Medetomidin (0.5 mg/kg BW; alfavet Tierarzneimittel GmbH, Neumünster, Germany) and dexamethasone (2 μg/g BW, Sigma-Aldrich, St Louis, MO, USA) was intraperitoneally administered before the surgery. Lidocaine (2%, AstraZeneca, Cambridge, UK) was applied subcutaneously before removing the scalp. A circular groove (3 mm in diameter) was made with a dental drill over the somatosensory cortex by repeated drilling. Then the skull was gently removed with forceps. Care was taken not to damage the dura. Standard extracellular solution (composition see above) was used to rinse the opening in the skull, which was then covered with a 3 mm glass coverslip (Warner Instruments, Hamden, CT, USA). The gap between the edge of the coverslip and the skull was filled with cyanoacrylate glue, and then strengthened by dental cement. A metal bar, which is needed for head fixation during two-photon imaging, was fixed to the skull posterior to the window. During the surgery, and until full recovery, the mouse was kept on a heating pad. Postoperative care included an analgesic dose of carprofen (5 μg/g BW, Pfizer, New York City, NY, USA) for three days, which was applied subcutaneously, and the antibiotic baytril (1:100 v/v; Bayer, Leverkusen, Germany) in drinking water for ten days. After the surgery, animals were allowed to recover for 3 weeks and were subsequently examined for window clarity and new bone growth.

### *In vivo* imaging

For *in vivo* imaging of microglia, the head of the mouse was fixed in the imaging setup by attaching either the recording chamber (acute experiments) or the metal holder (chronic experiments) to the stage of the microscope. Two-photon imaging was performed under isoflurane anaesthesia (0.8–1.3% in pure oxygen) using a laser-scanning microscope (Olympus Fluoview 1000, Olympus, Tokio, Japan) coupled to a mode-locked laser (MaiTai HP DeepSee, Spectra Physics, Santa Clara, CA, USA). Twitch-2B was excited at a wavelength of 890 nm. The emitted light was collected with either a 20x (1.0 NA, Zeiss, Oberkochen, Germany) or a 40x (0.80 NA, Nikon, Tokio, Japan) water-immersion objective and split into mCerulean3 and cpVenus^CD^ channels with a 515 nm dichroic mirror (filters used: 475/64 nm bandpass and 500 nm longpass). For monitoring of spontaneous Ca^2+^-transients in microglia, images were collected at a frame rate of 1 frame/s over a time period of 10 min. For imaging of drug-evoked and cell damage-induced Ca^2+^-transients, images were collected at a frame rate of 0.13 frames/s over a time period of 30–120 s. Image series were acquired at a spatial resolution of 0.15–0.31 µm/pixel. To estimate Ca^2+^-dependent alterations in fluorescence, a region of interest was drawn manually around microglial somata. The mean fluorescence intensity was determined for cpVenus^CD^ and mCerulean3 channel and ratios (R) were calculated by dividing background-subtracted fluorescence from both channels and expressed as relative change (ΔR/R). A change in ΔR/R was accepted as a Ca^2+^ transient if its amplitude was higher than three times the standard deviation of the background noise.

### Drug application

UDP (Uridine 5-diphosphate; Sigma-Aldrich, St Louis, MO, USA) and Bz-ATP (3′-O-(4-benzoyl)benzoyl ATP; Sigma-Aldrich, St Louis, MO, USA) were dissolved in standard pipette solution (150 mM NaCl, 2.5 mM KCl, 10 mM HEPES, pH 7.4) at a concentration of 1 mM. The drugs were locally applied to the microglial cells of interest using a pressure application system. Alexa Fluor 594 was routinely added to the drug solution at a concentration of 1 µM to improve visualization of the pipette. The pipette was positioned at a distance of 30–40 µm from the cell and a pressure pulse of 20–35 kPa was applied for 100–200 ms (applied volume: 11–176 pl, as estimated when using the same protocol to inject Alexa Fluor 594-containing dye solution into a block of 2% agar).

TTX (Tetrodotoxin; Biotrend, Köln, Germany) was added for 20 min to the extracellular solution used for perfusion of the recording chamber at a concentration of 2 µM.

### Cell damaging and laser-assisted tissue lesion procedures

The cell damaging procedure was performed in acute preparations as described previously^19, 22^. Briefly, a glass pipette filled with the standard pipette solution and Alexa Fluor 594 (100 µM) was positioned on the membrane of a neuron close (<50 µm) to the microglial cell of interest. The pipette was moved forward with a 10 µm step to damage the neuron while imaging the microglial Ca^2+^-signal. The time point of membrane rupture was estimated from an increase of Alexa Fluor 594 fluorescence within a region of interest located inside the soma of the damaged neuron. It should be noted that the cell damage-responsive microglial cell did not show any alterations in morphology and did not attract other microglial cells in their vicinity, suggesting that the procedure did not harm the microglial cells under study (see also ref. 19).

Laser-assisted tissue lesions were induced by exposing a confined square area of the brain parenchyma with dimensions of 63 × 63 µm or 79 × 79 µm located at a distance of 35–60 µm from the microglial cell of interest to transiently elevated intensities of laser light (890 nm wavelength, 3–4 s duration, 65 mW). Microglial cpVenus^CD^/mCerulean3 ratios were recorded directly before and 1–2 min after induction of the laser damage.

### Microglial cell culture

The primary microglial cell culture was prepared as described in refs 39 and 40 with slight modifications. Briefly, the brains of mouse pups (P2) were dissected and immediately stored in ice-cold Hank’s buffered salt solution (HBSS). Cortical tissue was separated, cut into small pieces and incubated in HBSS containing 0.03% Trypsin and 0.005% DNase. Tissue was triturated and passed through a 40 micron mesh cell strainer. The flow-through fraction was collected in microglia culture medium (Dulbecco’s Modified Eagle Medium with 10% FBS, 1% sodium pyruvate, 1% Penicillin/Streptomycin).

Cell suspension was centrifuged at 10,000 rpm for 10 min at room temperature, and the cell pellet was resuspended in microglia culture medium for cell counting. Cells were plated at a density of 1 * 10^5^ cells/cm^2^ in Poly-L-lysine pre-coated tissue flasks and left to develop *in vitro* with the culture medium refreshed the 5th day after preparation and every consecutive 3 days. Isolation and plating of primary microglia was done by gentle flask shaking at day 10 *in vitro*.

After 16–27 days in culture primary microglial cells were transduced with a miR-9-regulated vector driving the expression of Twitch-2B under the PGK or CMV promoter, which was diluted 1:100 or 1:200 in the culture medium. Cells were incubated in the presence of the viral particles for 48 hours. Afterwards, the medium containing viral particles was replaced by fresh culture medium (composition see above).

Two-photon imaging of Twitch-2B-expressing living cells was performed 7–9 days after transduction as described above (see *In vivo* imaging chapter). During imaging, cells were continuously perfused with pre-warmed extracellular solution (composition see above).

After imaging of cultured microglia, saline was carefully removed from the dish and 4% formaldehyde in PBS was added to fix the cells overnight at 4 °C or 4 h at room temperature. Afterwards, cells were rinsed three times using PBS and unspecific binding sites were blocked with 13% normal donkey serum (Dianova) in PBS containing 0.5% Triton X-100 (Sigma-Aldrich, St Louis, MO, USA) for 1–2 h at room temperature. Microglial cells were stained (overnight at room temperature) using the following primary antibodies: rabbit-anti-Iba1 (1:1000, Wako, Neuss, Germany), goat-anti-IL-1β (1:200 or 1:500; R&D Systems, Minneapolis, MN, USA), rat-anti-CD68 (1:1000; AbD SeroTec, Oxford, UK). Afterwards, cells were incubated (2 h at room temperature) with the Alexa Fluor 488- and Alexa Fluor 594-conjugated secondary antibodies (1:1000 or 1:3000; Invitrogen, Grand Island, NY, USA). Fixed microglial cells were imaged as described above (see Immunohistochemistry chapter). Basal ratios of Twitch-2B were only analysed in cells found in post hoc experiments to be Iba1 positive.

### Data analysis and statistics

Acquired images were processed and analysed offline using ImageJ and custom-made routines of the Igor Pro software (Wavemetrics, Lake Oswego, Oregon, United States).

IL-1β positivity of microglia was determined by analysing the co-localization of the IL-1β signal with the Twitch-2B or Iba1 signal using ImageJ. A cell was considered IL-1β positive when the IL-1β-specific fluorescence was at least 1.6 times higher than the background. For quantification of CD68 fluorescence, regions of interest were generated semi-automatically in the background-subtracted Iba1-channel and mean fluorescence of the background-subtracted CD68 signal was determined in these regions of interest with ImageJ.

Data are shown as box-whisker-plots with boxes drawn from 75th percentile (top) to 25th percentile (bottom) and whiskers representing 90th and 10th percentile. Statistical analyses were performed using the software on the VassarStats Statistical Computation Web Site (http://vassarstats.net/) and MATLAB (The Mathworks). Comparisons between two groups were conducted with the Mann-Whitney Test. Non-parametric Kruskal-Wallis test with post hoc Dunn-Sidak correction was used in the case of multiple comparisons. Differences were considered significant if p-values were below 0.05.

## Electronic supplementary material

Supplementary Information
