# Supplementary material for: A new approach for ratiometric in vivo calcium imaging of microglia
Source: Sci Rep. 2017 Jul 20;7:6030. doi: 10.1038/s41598-017-05952-3 (PMC5519759; doi:10.1038/s41598-017-05952-3)

Supplementary Information for

A new approach for ratiometric in vivo calcium imaging of microglia

Bianca Brawek*[1], Yajie Liang*[1], Daria Savitska[1], Kaizhen Li[1], Natalie Fomin-Thunemann[1], Yury Kovalchuk[1], Elizabeta Zirdum[1], Johan Jakobsson[2] & Olga Garaschuk[1]

[1]Institute of Physiology II, University of Tübingen, 72074 Tübingen, Germany

[2]Laboratory of Molecular Neurogenetics, Department of Experimental Medical Science, Wallenberg Neuroscience Center and Lund Stem Cell Center, Lund University, Lund 221 84, Sweden

*These authors contributed equally to this work

Supplementary Figure Legends

Supplementary Figure S1. Wavelength-dependent light scattering does not bias in vivo cpVenus^{CD}/mCerulean3 ratios measured at different focal depths. Scatter plot illustrating in vivo cpVenus^{CD}/mCerulean3 ratios measured in microglial cells at different focal depths in acute (black) and chronic (red) preparations. In both conditions, there was no correlation between the depth and the cpVenus^{CD}/mCerulean3 ratio (Spearman's correlation coefficient r=-0.243, p=0.252 for acute; r=-0.118, p=0.675 for chronic preparations).

Supplementary Figure S2. Choice of promoter has no effect on cpVenus^{CD}/mCerulean3 ratios measured in primary microglia. Box plot showing cpVenus^{CD}/mCerulean3 ratios measured in primary microglia transduced with LV.Twitch-2B.miR-9.T under control of either PGK (29 cells) or CMV (50 cells) promoter. The measured ratios are similar for both vectors (p=0.8103, Mann-Whitney test).

Supplementary Figure S3. Activation markers are upregulated in cultured microglia. (**a, b**) MIP images of microglial cells in a fixed brain tissue (**a**, 2-22 μm depth, step 1 μm) and a fixed primary microglial cell culture (**b**, 5-9 μm depth, step 1 μm) labelled with an anti-Iba1 (green, left) and anti-CD68 (red, middle) antibody, respectively. Merged images are shown on the right. (**c, d**) MIP images of microglial cells in a fixed brain tissue (**c**, 4-25 μm depth, step 1 μm) and a fixed primary microglial cell culture (**d**, 3-9 μm depth, step 1 μm) labelled with an anti-Iba1 (green, left) and anti-IL-1β (red, middle) antibody, respectively. Merged images are shown on

the right. (**e**) Box plot illustrating the distributions of background-subtracted CD68 fluorescence in microglial cells in fixed tissue (n=321 cells in 10 mice) and cell culture (n=210 cells in 5 dishes). Note that CD68 fluorescence is significantly higher in cultured microglia (p=0.003, Mann-Whitney test). (**f**) Box plot showing the fraction of IL-1β positive cells among microglial cells in fixed slices (n=10 mice) and cell culture (n=24 field of views in 3 dishes). Note that cultured microglia have a significantly higher fraction of IL-1β positive cells in comparison to microglia in the intact brain, where no IL-1β positivity was seen (p<0.001, Mann-Whitney test).

Supplementary Figure S1. Wavelength-dependent light scattering does not bias in vivo cpVenus^{CD}/mCerulean3 ratios measured at different focal depths

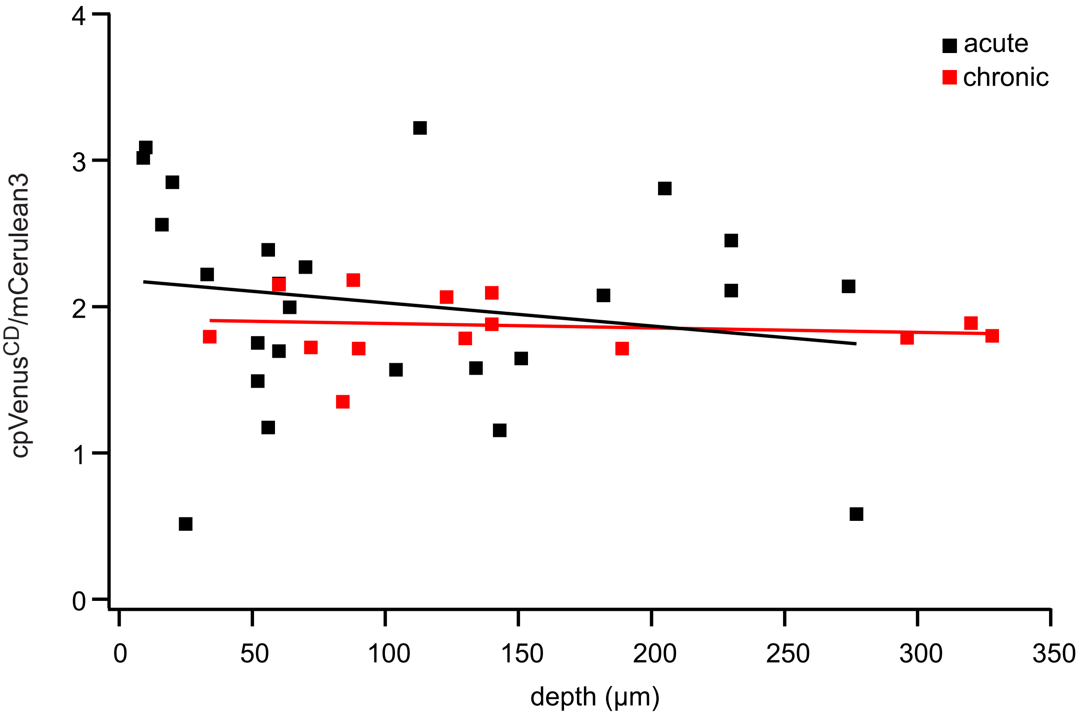

Supplementary Figure S2. Choice of promoter has no effect on cpVenus^{CD}/mCerulean3 ratios measured in primary microglia

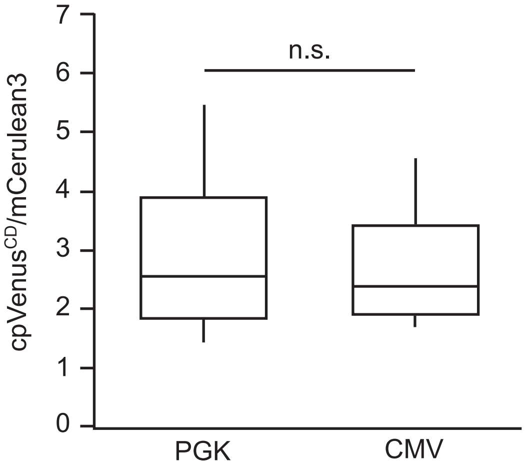

Supplementary Figure S3. Activation markers are upregulated in cultured microglia

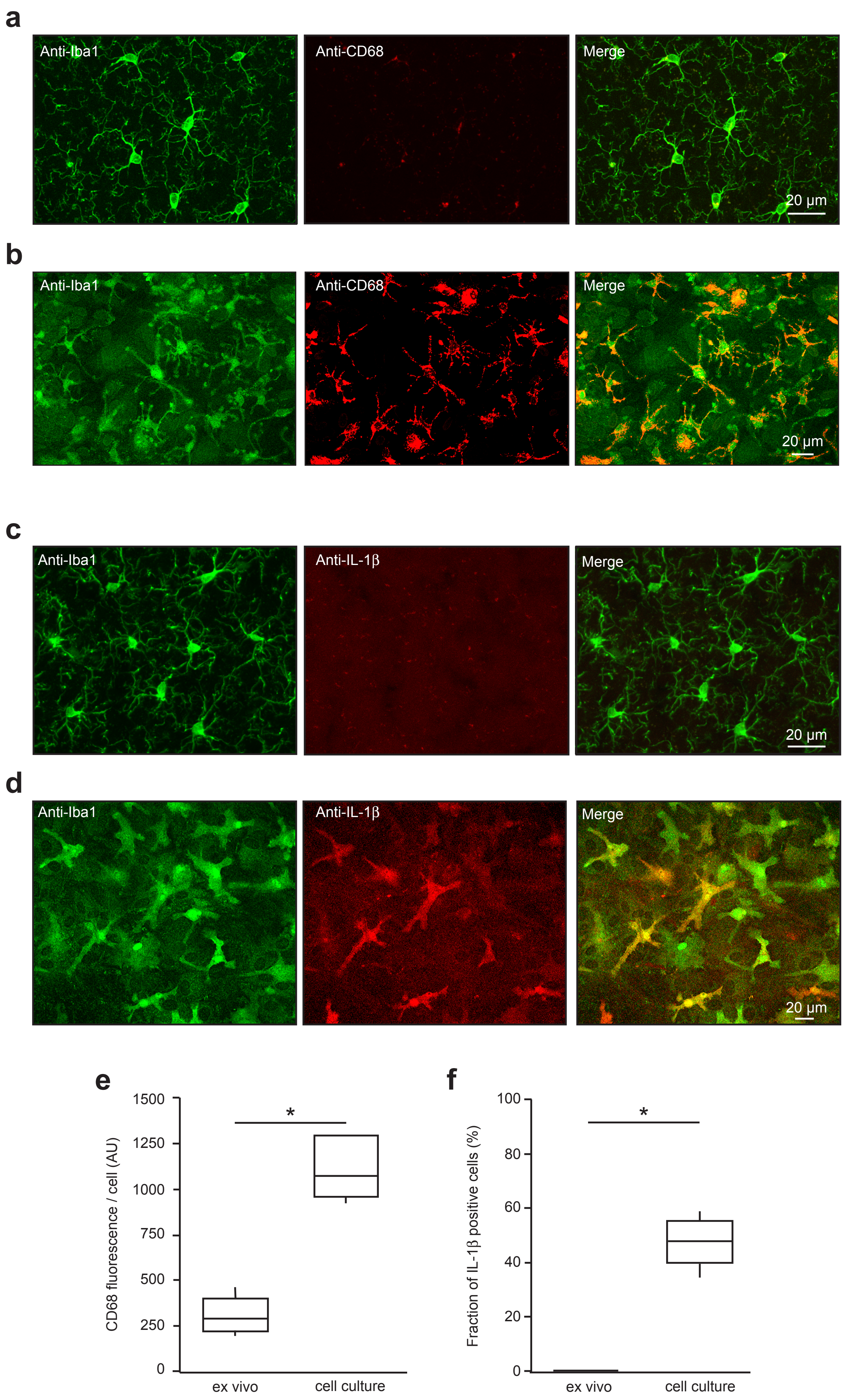

Supplement: Supplementary file 1 — Supplementary Information [file 41598_2017_5952_MOESM1_ESM.pdf]
